# Eosinophilic pustular folliculitis after hematopoietic stem cell transplantation: A study of 11 cases

**DOI:** 10.1111/1346-8138.15846

**Published:** 2021-03-14

**Authors:** Yu Sasaki, Akiko Kishi, Shinsuke Takagi, Naoyuki Uchida, Nobukazu Hayashi

**Affiliations:** ^1^ Department of Dermatology Toranomon Hospital Tokyo Japan; ^2^ Department of Hematology Toranomon Hospital Tokyo Japan

Dear Editor,

Eosinophilic pustular folliculitis (EPF) is classified into three subtypes: classic EPF (Ofuji’s disease), immunosuppression‐associated EPF (IS‐EPF), and infancy‐associated EPF. Recent studies have reported that IS‐EPF may occur in patients with HIV infection and those with hematological malignancies, particularly after hematopoietic stem cell transplantation (HSCT). However, few studies have investigated the association between the onset of IS‐EPF and the HSCT type.

Here, we examine 11 cases of IS‐EPF after HSCT, which were diagnosed by skin biopsies in our department between 2009 and 2019 (Table 1). This group comprised 10 men and one woman, with a median age of 50 years (range, 19–71). They presented with skin lesions appearing as scattered follicular papules, pustules, and post‐inflammatory pigmentations distributed predominantly over the upper part of the body, including the scalp and face. All patients had severe pruritus and histopathological evidence of eosinophilic follicular infiltration, and seven patients developed peripheral eosinophilia. Underlying hematological disorders included myelodysplastic syndrome (n = 3), acute myeloid leukemia (n = 2), acute lymphoid leukemia (n = 2), aplastic anemia (n = 2), multiple myeloma (n = 1), and angioimmunoblastic T‐cell lymphoma (n = 1), while the graft types included peripheral blood stem cells (PBSC; n = 2), bone marrow (BM; n = 3), and cord blood (CB; n = 6). CB recipients developed skin lesions 55–2261 days (median, 151) after transplantation, while PBSC and BM recipients developed skin lesions 6–64 days (median, 35) and 44–182 days (median, 49) after transplantation, respectively. The patients received oral indomethacin, topical indomethacin, or topical steroid, and nine patients completely resolved.

**TABLE 1:** Clinical characteristics of 11 patients with IS‐EPF after HSCT

	Age/sex	Graft type of HSCT	Onset after HSCT (days)	Underlying diseases	Location of skin lesions	Pruritus	WBC (/µL)	Eosinophils (/µL)	Lymphocytes (/µl)	Treatment	Outcome
1	55/F	PBSC	6	AITL	Scalp, trunk, upper and lower limbs	Yes	3300	297	578	Oral indomethacin	Resolved in 8 months
2	54/M	PBSC	64	MM	Scalp, face, trunk, upper limbs	Yes	4100	902	1210	Oral indomethacin, topical steroid	Resolved in 9 months
3	32/M	BM	44	MDS	Scalp, face, trunk	Yes	4700	1410	376	Oral indomethacin	Resolved in 2 months
4	45/M	BM	49	MDS	Trunk, upper limbs	Yes	5100	1071	1581	Oral indomethacin, topical steroid	Resolved in 3 months
5	50/M	BM	182	ALL	Scalp, face, trunk, upper limbs	Yes	4500	428	2138	Topical steroid, topical indomethacin	Incomplete response
6	24/M	CB	55	AML	Scalp, face, trunk, upper limbs	Yes	7300	4015	657	Oral and topical indomethacin	Resolved in 2 weeks
7	25/M	CB	80	AML	Scalp, face, trunk, upper limbs	Yes	10 800	2646	2106	Oral indomethacin, topical steroid	Resolved in 8 months
8	61/M	CB	137	AA	Trunk, upper and lower limbs	Yes	4600	437	966	Oral and topical indomethacin	Incomplete response
9	19/M	CB	165	AA	Scalp, face, trunk, lower limbs	Yes	5400	1539	1350	Oral and topical indomethacin	Resolved in 11 months
10	71/M	CB	502	MDS	Face, trunk, upper limbs	Yes	7000	560	2450	Oral and topical indomethacin, topical steroid	Resolved in 4 months
11	44/M	CB	2261	ALL	Face, trunk, upper and lower limbs	Yes	5900	679	1151	Oral indomethacin, topical steroid	Resolved in 2 months

Abbreviations: AA, aplastic anemia; AITL, angioimmunoblastic T‐cell lymphoma; ALL, acute lymphoid leukemia; AML, acute myeloid leukemia; BM, bone marrow; CB, cord blood; GVHD, graft‐versus‐host disease; HSCT, hematopoietic stem cell transplantation; IS‐EPF, immunosuppression‐associated eosinophilic pustular folliculitis; MDS, myelodysplastic syndrome; MM, multiple myeloma; PBSC, peripheral blood stem cell.

Follicular papules and pustules with severe and persistent itching predominantly involving the upper part of the body are the clinical characteristics of IS‐EPF, different from those of classic EPF. These clinical features, along with a history of HSCT and histopathological findings, led to a definitive diagnosis of IS‐EPF. Our results of IS‐EPF onset in PBSC and BM recipients are consistent with those of the previous studies,^1^, ^2^ which reported that skin lesions occur 2–3 months after PBSC or BM transplantation. Furthermore, our results demonstrated that skin lesions were likely to occur later in CB recipients than in PBSC or BM recipients. The greater delayed onset in CB recipients may be attributed to delayed immune reconstitution as the previous studies^3^, ^4^ showed significantly lower CD3^+^, CD4^+^, and CD8^+^ T cells in CB recipients than in PBSC or BM recipients until 6 months after transplantation. It is also noteworthy that HIV patients develop IS‐EPF as an immune reconstitution inflammatory syndrome (IRIS) during the increasing number of CD4^+^ T cells 3–6 months after initiation of antiretroviral therapy.^5^ These data suggest that skin lesions of IS‐EPF occur during the period of immune reconstitution both in patients with HIV infection and in patients after HSCT. These patients’ common immune status featured cellular immunodeficiency with an inverse CD4/CD8 ratio caused by delayed T‐cell reconstitution. The limitations of this study are its small sample size and retrospective design. Further accumulation of cases and prospective studies are required to confirm the association between IS‐EPF and IRIS.

## CONFLICT OF INTEREST

None declared.
